# Identification of Potential Allosteric Site Binders of Indoleamine 2,3-Dioxygenase 1 from Plants: A Virtual and Molecular Dynamics Investigation

**DOI:** 10.3390/ph15091099

**Published:** 2022-09-02

**Authors:** Vitor Martins de Almeida, Osvaldo Andrade Santos-Filho

**Affiliations:** Laboratório de Modelagem Molecular e Biologia Estrutural Computacional, Instituto de Pesquisas de Produtos Naturais Walter Mors, Centro de Ciências da Saúde, Universidade Federal do Rio de Janeiro, Av. Carlos Chagas Filho, 373, Bloco H, Cidade Universitária, Rio de Janeiro 21941-599, RJ, Brazil

**Keywords:** cancer, immunology, flavonoids, IDO1, virtual screening, molecular docking, molecular dynamics, free energy

## Abstract

Ligand and structure-based computational screenings were carried out to identify flavonoids with potential anticancer activity. Kushenol E, a flavonoid with proven anticancer activity and, at the same time, an allosteric site binder of the enzyme indoleamine 2,3-dioxygenase-1 (IDO1), was used as the reference compound. Molecular docking and molecular dynamics simulations were performed for the screened flavonoids with known anticancer activity. The following two of these flavonoids were identified as potential inhibitors of IDO1: dichamanetin and isochamanetin. Molecular dynamics simulations were used to assess the conformational profile of IDO1-flavonoids complexes, as well as for calculating the bind-free energies.

## 1. Introduction

### 1.1. Tryptophan Metabolism and the Kynurenine Pathway

Studies show that tumor tissues within a chronic inflammatory context (presence of pro-inflammatory cytokines for a long time) catabolize in large proportions the amino acid L-tryptophan in the cell matrix and synthesize metabolites from one of the main degradation pathways of tryptophan, the pathway of kynurenine [1,2].

**Figure 1:**
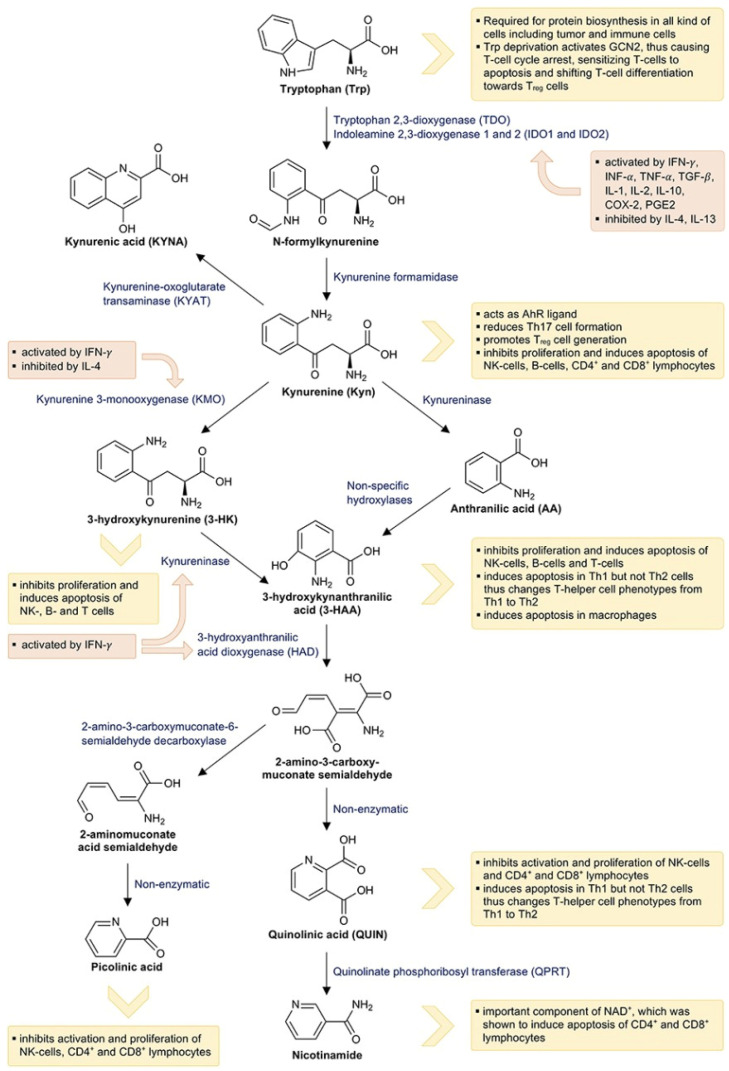
Catabolization of tryptophan via the kynurenine pathway and its interactions with the immune system. Orange boxes indicate the effects of immune mediators on the kynurenine pathway and yellow boxes indicate the effects of tryptophan metabolites on the immune system. Adapted from Lanser et al. [4].

Almost all metabolites of the kynurenine pathway affect immune activity through various mechanisms. Figure 1 illustrates the effects of kynurenine pathway metabolites on the immune system. The determining step of the kynurenine pathway is the formation of N-formylkynurenine, regulated by the family of dioxygenase enzymes known as tryptophan 2,3-dioxygenase (TDO) and indolamine 2,3-dioxygenase (IDO), which may exist in the following two isoforms: IDO1 and IDO2 [3].

TDO, IDO1, and IDO2 are heme-bearing enzymes and catalyze the rate-limiting step of tryptophan catabolization. TDO is mainly expressed in the liver and oxidizes excess tryptophan in the body, generating ATP and especially NAD+. The expression of TDO is stimulated by the concentration of blood tryptophan as well as by the synthesis of the heme cofactor [4].

IDO1 can be expressed by many different cells, including antigen-presenting cells (APCs), such as monocyte-derived macrophages, dendritic cells (DCs), and fibroblasts. Its expression is mainly induced by inflammatory cytokines, such as IFN-γ, TNF-α, IL-1, and IL-2, secreted by Th1-type lymphocytes, as well as TGF-β, IL-10, and adenosine secreted by regulatory T lymphocytes [5]. IDO1 expression is further stimulated by its own product, kynurenine, via the aryl hydrocarbon receptor (AhR) [6,7].

IDO2 is significantly less active than IDO1 [8]. Similar to IDO1, its expression is stimulated by the activation of AhR [9]. Although IDO2 is expressed by cancer cells, when compared to IDO1, it contributes little to the accumulation of kynurenine pathway metabolites [10,11].

In healthy patients, IDO1 expression is restricted to endothelial cells in the placenta, lung, mature dendritic cells in secondary lymphoid organs, and in epithelial cells scattered in the female genital tract [12]. Whereas, under inflammatory conditions, IDO1 expression is strongly induced by INF-γ [13]. Furthermore, it is highly expressed at inflammatory sites, where it may contribute to negative feedback against local immune response activity [12]. Inflamed tumors may also express IDO1 as an adaptive resistance mechanism, in which it is induced by IFN-γ produced by dendritic cells and tumor-infiltrated macrophages [14].

Given the physiological importance of the IDO1 enzyme within the tumor microenvironment, the need for structural studies and the development of increasingly efficient inhibitors has become an attractive approach to treating cancer patients.

### 1.2. IDO1: Structure

**Figure 2:**
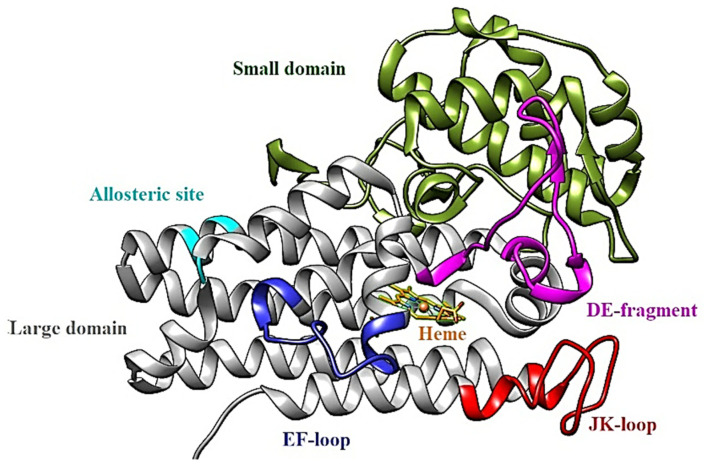
Structure of indoleamine deoxygenase 1 (IDO1) with highlighted regions: minor domain (green) and major domain (grey, magenta, red, blue, and cyan). Contained in the larger domain are: the DE fragment, composed of the DE hairpin and the DE loop; JK loop; EF loop; the heme cofactor (orange); the allosteric site.

The human IDO1 is a monomeric α-helical dioxygenase enzyme containing an N-terminal minor domain (residues 1–154) and a C-terminal major domain (residues 155–403), where the catalytic site and a cofactor heme are located (Figure 2).

The minor domain or N-terminal domain, shown in green in Figure 2, contains residues Gly11 to Asp158, consisting of six α-helices (involving amino acids Tyr36 to His45, His45 to Ser52, Gln54 to Lys61, Asp72 to Gly93, Pro104 to Glu119 and Val125 to Val130 or helix A, contributing to the active site), four 3_10_-helices (Ser12 to His16, Pro33 to Phe35, Ser66 to Leu70 and Thr144 to Glu146) and a small antiparallel β-sheet formed by residues Lys135 to Lys136 and Met148 to Asp149. Such a domain makes up part of the upper region of the catalytic site and acts as structural support for the enzyme [15].

The major domain or C-terminal domain, shown in grey in Figure 2, contains residues Cys159 to Gly403, being constituted by 9 α-helices [15]. This domain performs functions related to conversion, positioning, and displacement of substrates/products in the enzyme and is where the catalytic site is located. There are also regions with specific functionalities that are important for the enzymatic reaction to occur. Among them, there is the “access” tunnel for the entrance of small molecules from the external environment (ligand delivery tunnel) and the JK loop (red), with the function of conducting and maintaining the substrate (tryptophan) inside the catalytic site [16].

Finally, there is the DE fragment, consisting of the DE-hairpin (Ser235 to Tyr249) and the DE loop (Glu250 to Gly262) (magenta), the EF loop (Gly278 to His287) (blue), and the JK loop (Gln360 to Gly380) [17].

IDO1 has the following two non-competitive natural substrates: tryptophan and molecular oxygen (O_2_). Moreover, the enzyme has the following four states:

- apo-IDO1: without the heme cofactor;
- holo-IDO1: containing the iron atom of the heme group in the ferric state (Fe^3+^);
- holo-IDO1 with the iron atom of the heme group in the ferrous state (Fe^2+^);
- O_2_-linked holo-IDO1 complex.

Despite not being the active form of the enzyme, apo-IDO1 is a promising macromolecular target since cellular studies have shown that this form is the most abundant in the cell medium, about 85% [18]. Therefore, it is likely that there is a binding site where potential inhibitors of the apo form can interact with this form of the enzyme. The main objective of this project was to apply molecular modeling methods in order to investigate this possibility.

### 1.3. IDO1: Inhibitors

Over the past decade, the scientific academy has been making efforts for the development of IDO1 inhibitors [2]. Röhrig and collaborators classified existing test-phase inhibitors into the following four categories: type I, II, III, and IV inhibitors [19].

Type I inhibitors are classified as competitive inhibitors of tryptophan. In general, type I inhibitors target the molecular oxygen-bound holo-IDO1 form and do not form a direct bond with heme iron [17]. The 1-LMT and 1-DMT are classified within this category of inhibitors [20].

Type II inhibitors are classified as competitive inhibitors of molecular oxygen by binding to the iron atom in its ferrous state of the heme group, acting in the ferrous holo-IDO1 form. Inhibitors such as β-carboline [21], and Epacadostat [22] fall into this category.

Type III inhibitors are noncompetitive inhibitors of tryptophan. New inhibitors in this class are being studied, such as 4PI, Navoximod, and MMG-0358 [19].

Type IV inhibitors are the most recently described class of inhibitors in the literature, including inhibitors such as the clinical compound BMS-986205, which targets the apo-IDO1 form [23].

**Figure 3:**
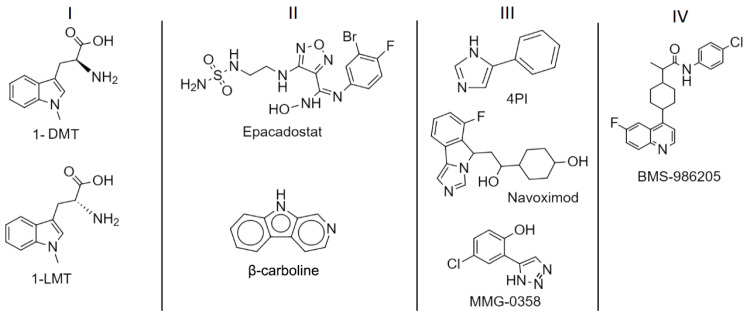
IDO1 inhibitors.

The structures of the mentioned inhibitors are shown in Figure 3.

**Figure 4:**
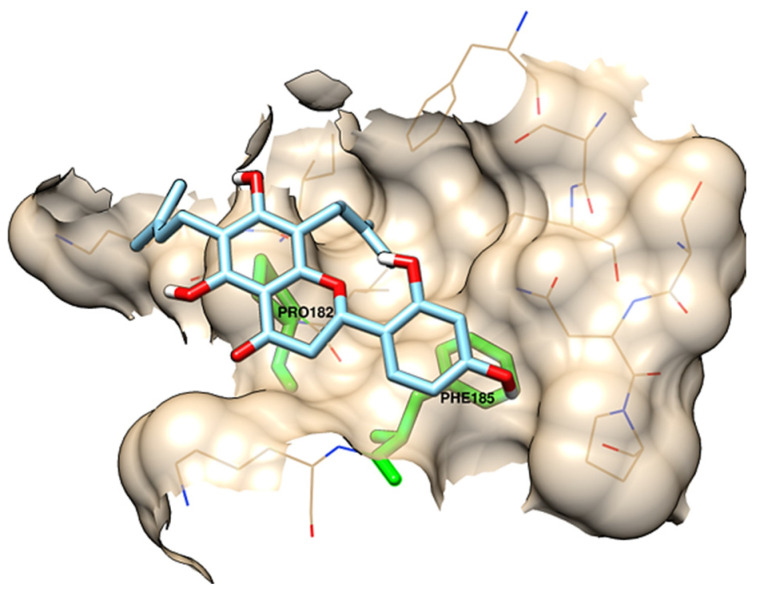
Allosteric site described by Kwon and colleagues [24]. The docked flavonoid Kushenol E, as well as Pro182, and Phe185 are shown in detail.

Kwon and collaborators [24] determined the amino acid residues that define an allosteric site for the action of apo-IDO1 inhibitors (Ser12, Tyr15, Ile17, Ile178, Lys179, Ile181, Pro182, Phe185, Lys186, Phe306, Ser309, Leu310, Ser312, Asn313, and Pro314), and found that the flavonoid obtained from plants of the species *Sophora flavescens*, known as Kushenol E, acts as an inhibitor of this site (Figure 4). The authors described the amino acids Pro182 and Phe185, located in the larger domain of the enzyme, as the main components of the allosteric site for inhibitory activity. Site-directed mutagenesis experiments were performed on these residues, and in vitro enzyme inhibition assays were performed. The results proved the contribution of these amino acids to enzymatic function and indicate this allosteric site as a target for new inhibitors [24].

Most IDO1 inhibitors are obtained from a natural source; however, as shown by Kwon, flavonoids may inhibit IDO1 [24]. The diverse variety of natural products can make the screening of these compounds a time-consuming and expensive task. Due to the development of more powerful computers, virtual screening of chemical compounds and pharmacodynamic validation of ligand-receptor interactions can accelerate the process of discovering potential new drugs. In this context, we focused our work on the identification of potential inhibitors of the naturally occurring apo-IDO1 allosteric site.

## 2. Results and Discussion

### 2.1. Ligand-Based Virtual Screening

**Figure 5:**
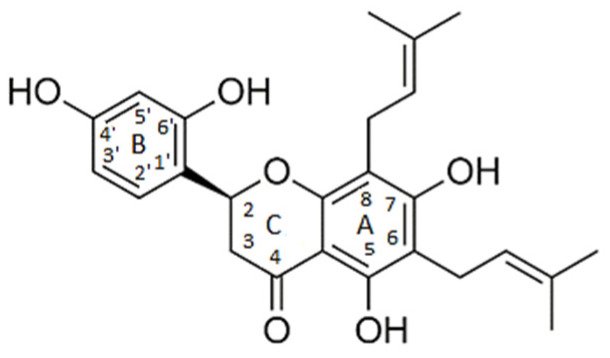
Kushenol E.

In order to identify potential IDO1 inhibitors capable of interacting with its allosteric site, the following two virtual screening tools were used: the Mcule platform [25] and the ZINC 15 database [26]. At this stage of the work, the flavonoid Kushenol E (Figure 5) was used as the reference compound, due to its proven effectiveness as a non-competitive inhibitor that binds to the allosteric site of the enzyme [24]. Tanimoto’s coefficient was used as the sampling criterium [27], and 172 compounds were sampled.

### 2.2. Structure-Based Virtual Screening and Molecular Docking

**Figure 6:**
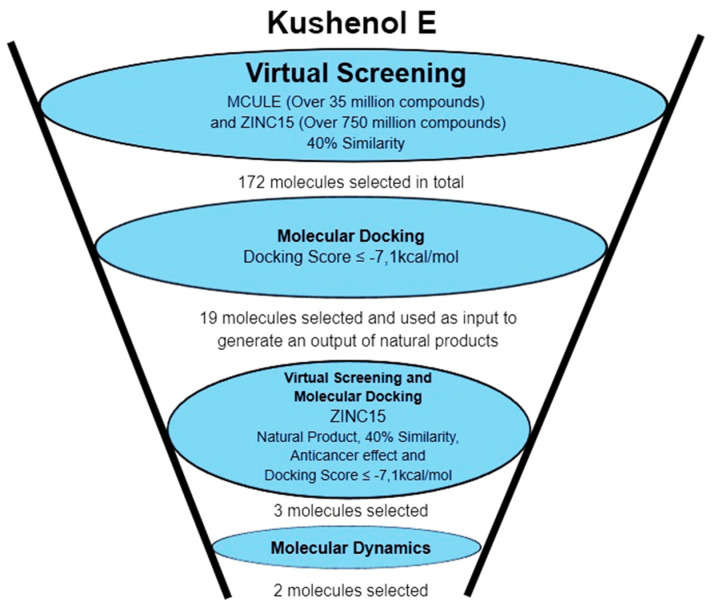
Workflow of ligand-based and structure-based virtual approaches.

Besides the ligand-based virtual screening mentioned above, a structure-based virtual screening was also carried out (Figure 6). In this approach, the previously sampled compounds, including Kushenol E, were docked into the allosteric site of the IDO1.

The calculated docking energies of each compound are shown in Supplementary Material (Table S1). The range of calculated energies varied from −8.1 kcal/mol to −5.3 kcal/mol. The docking energy for Kushenol E is equal to −6.6 kcal/mol. We realized that most of the sampled compounds were not from natural sources. Since the focus of this research was the identification of substances obtained from natural products with the potential ability to inhibit the biological response of the enzyme IDO1 through interaction at the allosteric site, a new virtual screening was carried out. The strategy was to use each compound that presented interaction energies equal to or lower than −7.1 kcal/mol (19 compounds; represented in red in Supplementary Materials, Table S1) as reference compounds in new ligand-based virtual screenings. The ZINC 15 database was the chosen tool. In this context, 28 natural products were selected, which were later docked to the allosteric site of the IDO1 enzyme. The result of this second-round structure-based virtual screening is shown in Table 1. While, in this investigation, Kushenol E (shown in bold red) works as the positive control, it is also important to consider a compound that would work as a negative control. In this context, as in the paper by Kwon [24], steppogenin was chosen as such a compound. As shown in the table, among all compounds, steppogenin shows the least favorable docking energy (shown in light red).

In this second molecular docking calculation, two additional criteria were used for the final screening as follows: (a) compounds that presented docking energies equal to or lower than −7.1 kcal/mol; (b) compounds that showed some anticancer activity (as described in the literature). Consequently, the following three flavonoids were selected: dichamanetin, isochamanetin, and chamaejasmin B, shown in bold black.

Dichamanetin is a C-benzylated flavanone that can be obtained from several Southeast Asian plant species, such as the following: *Uvaria alba*, *Uvaria chamae*, *Piper sarmentosum*, and *Xylopia pierrei* [28,29,30]. Isochamanetin, another C-benzylated flavanone, was obtained from several plant species such as *Sphaeranthus amaranthoides*, *Xylopia pierrei*, and *Ulvaria chamae* [28,31,32]. Chamaejasmin B is a dimeric biflavonoid formed by two isosacuranetin subunits linked by C3-C3 carbons, and obtained from Southwest Asian plant species, such as *Stellera chamaejasme L*. [33].

**Figure 7:**
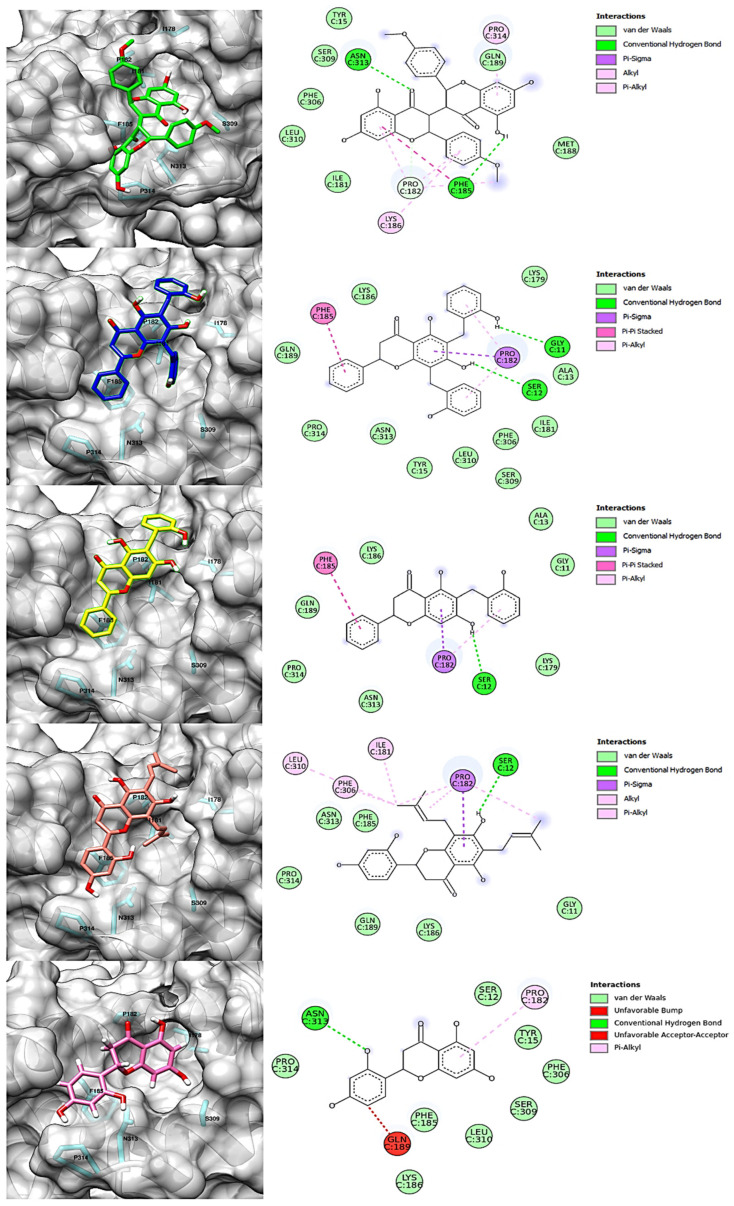
Intermolecular interactions formed with specific residues from the allosteric site of IDO1: chamaejasmin B, dichamanetin, isochamanetin, Kushenol E, and steppogenin, respectively.

Table 2 and Figure 7 show the intermolecular interactions formed with specific residues of the allosteric site of IDO1.

As shown in Table 2 and Figure 7, the flavonoids interact with amino acid residues from the allosteric site, as defined by Kwon [24] (Ser12, Tyr15, Ile17, Ile178, Lys179, Ile181, Pro182, Phe185, Lys186, Phe306, Ser309, Leu310, Ser312, Asn313, Pro314). Since flavonoids are compounds that have several aromatic rings, the main interactions formed with the enzyme are of the pi and van der Waals types. The screened flavonoids (Table 2) have a significant number of oxygens; consequently, they can work as hydrogen bond donors and acceptors. Furthermore, according to Kwon [24], residues Pro182 and Phe185 are essential for the structural and functional integrity of the IDO1 enzyme system.

Dichamanetin and isochamanetin A rings interact via pi-sigma interaction with Pro182. According to Kwon [24], this residue would be important for the structural and functional integrity of the IDO1 system. In chamaejasmin B, which is a biflavonoid, one of the A rings interacts with Pro182 (pi-alkyl) and Phe185 (pi-pi) with the assistance of van der Waals interactions with neighboring residues; the second ring A interacts with Phe185 (hydrogen bond) and with Pro314 (pi-alkyl), in addition to van der Waals interactions. 

Focusing on substituents attached to the A ring of flavonoids, some structural features must be considered. The hydroxyl group attached to carbon 7 of the A rings of isochamanetin, dichamanetin, and Kushenol E forms strong hydrogen bond interactions with Ser12. Phenyl groups linked to the A ring of dichamanetin and isochamanetin probably help in the stability of the interaction of these compounds with the allosteric site. As shown in Table 2, dichamanetin interacts more strongly with IDO1 than with isochamanetin (−7.8 kcal/mol and −7.5 kcal/mol, respectively), with the first compound forming two pi-alkyl interactions with Pro182. It is also noted that the participation of a weak hydrogen bond interaction with Gly11. Isochamanetin, on the other hand, forms only one interaction. As will be shown in the next section, referring to molecular dynamics simulations, both Ser12 and Pro182 residues contribute significantly to the energetic stabilization of the formed complex.

Rings B of both dichamanetin and isochamanetin interact via pi-pi stacking with Phe185. Ring B of chamaejasmin B interacts with Pro182 and Lys186 (pi-alkyl interaction). The methoxyl group attached to the C4’ carbon of the ring B of chamaejasmin B forms an alkyl interaction with Pro182. Of all the flavonoids, the only one that interacts with IDO1 from the C ring is chamaejasmin B. This occurs through a hydrogen bond with a carbonyl group on carbon 4 of the C ring. It is worthy of note the fact that steppogenin is the only compound that shows unfavorable bump and acceptor-acceptor interactions.

**Figure 8:**
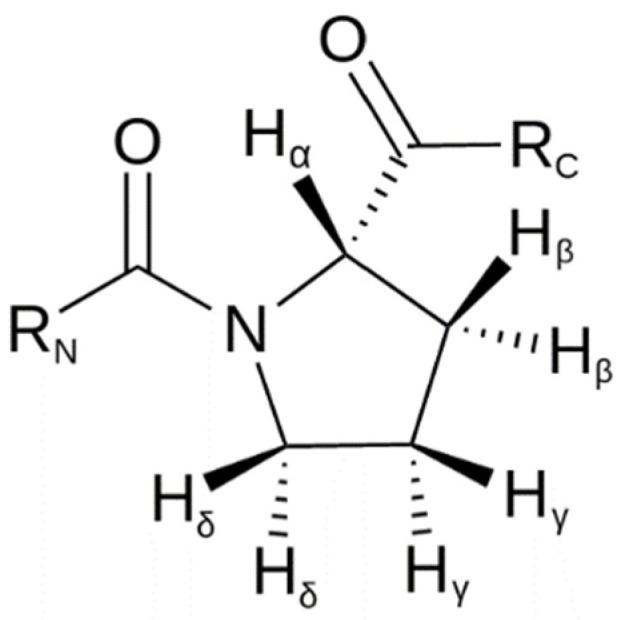
Hydrogens in proline.

Pro182 interacts through pi interactions or with the A ring of flavanones or with phenolic groups attached to the same ring. It is known that the proline ring faces are partially positively charged [34]. As shown in Figure 8, hydrogens adjacent to the carbonyl and nitrogen of the amide (Hα and Hδ, respectively) are the most partially positive. The proline side chain is also conformationally restricted, allowing interaction with aromatic residues with minimal entropic or steric penalty [34].

**Figure 9:**
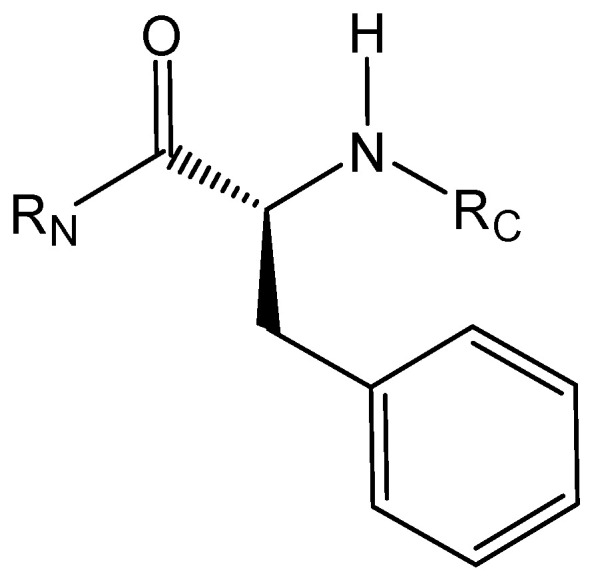
Hydrogens in phenilalanine.

Phe185, an aromatic residue (Figure 9), interacts with flavonoids through pi-pi and pi-pi/T stacking, and van der Waals interactions [35], which are very important for the stabilization of the structural energy of protein complexes with flavonoids.

**Figure 10:**
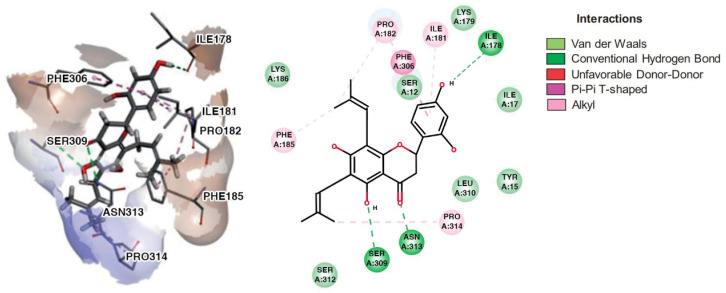
Kwon’s docking model of Kushenol E—IDO1 complex [24].

Comparing our docking modeling of Kushenol E (Figure 7) with the docking modeling proposed by Kwon [24] (Figure 10), we can see that our simulation shows this compound in a different orientation in the cavity of the allosteric site, with a deviation of the longitudinal axis towards the B direction for the prenyl group attached to the C6 carbon of the A ring. According to Kwon, the B ring of the flavonoid is positioned inside the cavity formed by the amino acids Leu178, Ile181, and Phe306 (Figure 10). Differently, we found the same ring located next to residues Leu178, Ile181, and Phe306. Furthermore, according to Kwon, A and C rings interact with Ser309 and Asn313. Instead, in our model, this interaction takes place with the Pro182, and there is assistance from a few van der Waals interactions. The Kwon model shows the prenyl group attached to carbon 6 of the ring interacting with Pro314, whereas our model shows interaction with Pro182. Since the structure of the IDO1-Kushenol E complex has not been experimentally determined to date, a definitive answer about the correct orientation of Kushenol E will only be inferred after the structural elucidation study by X-ray crystallography, nuclear magnetic resonance, or cryo-electron microscopy.

### 2.3. Molecular Dynamics Simulations and Free Energy Calculation

To further refine and investigate the ability of molecular docking simulation to predict the conformational profile of the IDO1-flavonoid complexes, and to calculate the respective binding free energies, molecular dynamics simulations were carried out.

**Figure 11:**
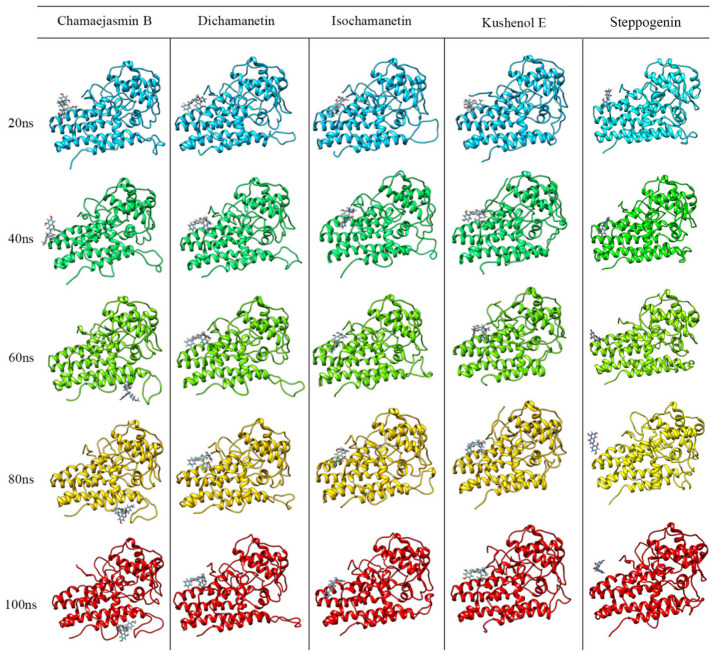
Conformational profile of the complexes formed by the interaction of IDO1 with chamaejasmin B, dichamanetin, isochamanetin, Kushenol E, and steppogenin, respectively, up to 100 ns molecular dynamic simulations.

Three-dimensional structures of the IDO1-flavonoid complexes at 20 ns, 40 ns, 60 ns, 80 ns, and 100 ns are shown in Figure 11. With the exception of chamaejasmin B, which detaches from the allosteric site cavity after 40 ns, all other flavonoids remained docked. Interestingly, after detaching from the allosteric site, chamaejasmin B stabilizes next to the JK loop. Furthermore, only one of the isosacuranetin subunits interacts with the allosteric site, while the other subunit is exposed to the solvent. Moreover, Figure 11 also shows that after 80 ns, steppogenin is almost completely detached from the allosteric site.

**Figure 12:**
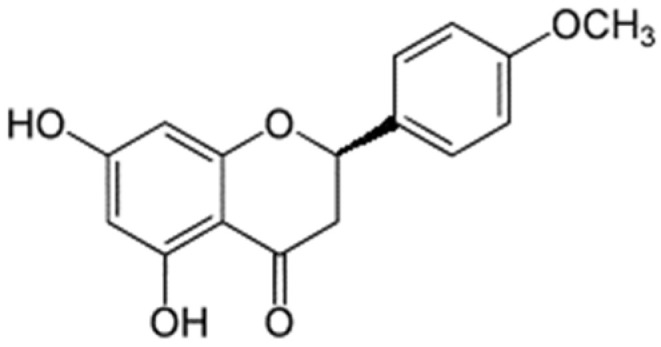
Isosacuranetin.

It is worthy of note that although the interactions of chamaejasmin B were not strong enough to keep it docked in place during 100 ns, this flavonoid showed the “best” docking energy. It is possible that its isosacuranetin subunit (Figure 12) be a good candidate for future studies of docking and molecular dynamics.

In order to verify how flavonoids could alter the conformational profile (packing/folding) of IDO1, radii of gyration (Rg) plots were calculated not only for IDO1 in its apo form but also for each IDO1-flavonoid complex. The corresponding plots are shown in Supplementary Material (Figure S1). In molecular dynamics, the radius of gyration is a measure of the stability of a system consisting of several particles and describes the variation in density as a function of the center of mass over time. According to Figure S1, the IDO1-steppogenin complex is the one with the highest degree of energetic-structural destabilization when compared to the other complexes. The IDO1-dichamanetin complex was the most stable. These stability data are supported by the values of relative molecular docking energies, shown in Table 1, and by the values of free energy of interaction between the flavonoids and IDO1, shown in Table 3.

Based on Figure S2, one can assess how each flavonoid changes the conformation of IDO1 (its apo conformation). In this context, Kushenol E and isochamanetin distort the IDO1 structure more significantly. Whereas dichamanetin is the flavonoid that less intensely distorts IDO1.

In addition to the radius of gyration and RMSD plots, root means square fluctuation (RMSF) for Cα of each enzyme residue was calculated (Figure S3). This measure is described by means of a two-dimensional graph where the abscissa shows each Cα associated with a given residue and the ordinate shows the average value of the fluctuations. It is verified that helices B (residues Cys159 to Met190) and G (residues Pro300 to Asn313), important for the formation of the allosteric site, do not undergo significant fluctuations due to the interaction of flavonoids. The EF loop (residues Gly278 to His287), significatively fluctuates due to the interaction of Kushenol E, steppogenin, and isochamanetin. Such an effect is less significant when dichamanetin is the interacting flavonoid. As mentioned before, residues between 251 and 300 and the EF loop are part of the molecular oxygen access tunnel structure. Figure S3 indicates that, of the three flavonoids, only dichamanetin does not “stiffen” the tunnel. The JK loop did not fluctuate significantly under the action of flavonoids.

RMSF plots were also constructed for each docked flavonoid into the allosteric site of IDO1 along with the molecular dynamic simulations (Figure S4). As can be seen, dichamanetin and isochamanetin showed lower atomic fluctuations than Kushenol E and steppogenin do. These results reinforce, once again, the relative stability of the interactions of each flavonoid with the allosteric site.

The complexes were also analyzed for the frequency of hydrogen bonds formed during the molecular dynamics simulation (Figure S5). Dichamanetin has a higher frequency of hydrogen bonds than isochamanetin and Kushenol E. These data demonstrate that hydrogen bonds are important for the stabilization of the IDO1-flavonoid complexes, mainly for dichamanetin and isochamanetin. However, steppogenin shows an “anomalous” hydrogen bond frequency. Perhaps that should be due to the highest degree of freedom (“pose fluctuations”), since, as shown in Figure 11, that compound seems to be slightly bound to the allosteric site.

The MM-PBSA method evaluates conformational fluctuations as a function of the simulation trajectory and, consequently, it is a useful approach for inferring the entropic profile of the formed complexes, as well as for calculating the free energy of binding of each flavonoid with the IDO1. The results are shown, respectively, in Table 3 and Table 4. According to Table 3, the affinity of each flavonoid with IDO1 increases in the following order: steppogenin (−5.73 kcal/mol); Kushenol E (−21.65 kcal/mol); isochamanetin (−22.09 kcal/mol); dichamanetin (−25.93 kcal/mol). It is worth noting the calculated affinity value for steppogenin (the negative control), testifying once again, and in accordance with the experimental data [24], that steppogenin forms the least stable complex with IDO1. The free energy contributions of each constituent residue of the allosteric site are shown in Table 4.

## 3. Materials and Methods

### 3.1. IDO1

The crystallographic structure of IDO1 in its holo form, with a resolution of 2.44 Å (PDB ID: 7A62) [15], was obtained from the Protein Data Bank [36]. The enzyme preparation steps were carried out as follows: (1) non-essential water molecules were removed; (2) polar hydrogens were added to the enzyme; (3) partial charges were calculated using both Kollman and Gasteiger’s approaches [37,38].

### 3.2. Virtual Screening

For the virtual screening of compounds, Mcule platform [25], ZINC 15 database [26], and AutoDock Vina 1.1.2 program [39] were used.

### 3.3. Molecular Docking

Molecular docking simulations were performed with the AutoDock Vina 1.1.2 program [39]. This tool uses a hybrid scoring function, i.e., it is a combination of empirical and knowledge-based functions [39]. The binding energy is predicted as the sum of distance-dependent atom pair interactions as follows:

$$E=\sum^{\;}e_{pair}\;\left(d\right)$$

Here *d* is the surface distance calculated as follows, where *r* is the interatomic distance and *R_i_* and *R_j_* are the radii of the atoms in the pairs:

$$d=r-R_{i}-R_{j}$$

Every atom pair interacts through a steric interaction given by the first three terms of the equation shown below. Moreover, depending on the atom type, there could be hydrophobic and non-directional H-bonding interactions, given by the last two terms.

$$e_{pair}(d)=\{\begin{array}{l} \;+\;\;w_{1}*\;Gauss_{1}\left(d\right)\\ \;+\;\;w_{2}*\;Gauss_{2}\left(d\right)\\ \;+\;w_{3}*\;Repulsion\left(d\right)\\ \;+\;w_{4}*\;Hydrophobic\left(d\right)\\ \;+\;w_{5}*HBond\left(d\right) \end{array}$$

The combination of an attractive Gaussian function with a repulsive parabolic function reproduces the general shape of a typical Lennard-Jones interaction, provided the Gaussian term is negative and the parabolic positive.

### 3.4. Molecular Dynamics Simulations and Free Energy Calculation

All molecular dynamics simulations were performed with the CHARMM36 force field [40] implemented in the GROMACS 2018.1 program [41]. The parameterization of flavonoids was performed using the CHARMM General Force Field (CGenFF) program [42]. Subsequently, a dodecahedral simulation box, including solvent inside (TIP3P water model) was created. Sodium and chlorine ions were added to the system, aiming to electronically neutralize it. Periodic boundary conditions were used.

Each complex was submitted to energy minimization using the steepest descent algorithm, involving 50,000 steps and the convergence criterion of less than 2.39 kcal/mol. Subsequently, two 100 ps molecular dynamics simulations were performed, aiming to balance the IDO1-flavonoid complexes. In the first simulation, the NVT ensemble was used, and in the second one, the NPT ensemble was used. In both cases, the simulation temperature was kept constant at 300 K and, when performing the NPT ensemble, the pressure was kept constant at 1 bar.

After the equilibration of the complexes, 100 ns molecular dynamics simulations were performed, aiming to calculate the free energies of interaction of each flavonoid with IDO1 (production stage). The running conditions were the following: ensemble NPT, where temperature was maintained, using the V-rescale implementation of Berendsen’s thermostat [43]. A molecular frame was sampled every 10 ps. To keep the pressure constant, the Parrinello-Rahman pressure coupling method was used [44]; for the long-range treatment, the PME method was used [45].

Free energy calculations were calculated with the g_mmpbsa tool [46]. The binding energy can be individually decomposed as a function of the constituent residues of the macromolecular target. Initially, the E_MM_, G_polar,_ and G_nonpolar_ energy components of the individual atoms of each residue are calculated in the bound, as well as the unbound form. Subsequently, the contribution to the interaction energy $\Delta R_{x}^{BE}$ of the residue *x* is calculated as follows:

$$\Delta R_{x}^{BE}=\sum_{i=0}^{n}\left(A_{i}^{bound}-A_{i}^{free}\right)$$

where, $A_{i}^{bound}$ and $A_{i}^{free}$ are the energy of atom *i* of residue *x* in bonded and unbonded states, respectively, and *n* is the total number of atoms in the residue. The energy contribution added to all residues is equal to the interaction energy, that is, $\Delta G_{binding}=\sum_{x=0}^{m}\Delta R_{x}^{BE}$, where *m* is the total number of residues that make up the ligand-protein complex [46].

### 3.5. Visualization Tools and Plots

Molecular Visualization Programs BIOVIA Discovery Studio 2021 [47], UCSF Chimera [48], and AutoDock Tools [49] were used. Grace plotting tool was used for graphical analysis [50].

## 4. Conclusions

IDO1, one of the enzymes that participate in the immune modulation process, is an interesting macromolecular target for anticancer action. From a known drug (Kushenol E), with proven anticancer activity, extensive virtual screening studies were carried out in digital databases. Both ligand- and structure-based approaches were performed. In this context, three natural products, isolated from plants, were identified as potential allosteric site binders of IDO1. Two of which, dichamanetin and isochamanetin, were shown to be promising IDO1 inhibitors and, in our opinion, should be considered in future studies of biological activity, molecular optimization, organic synthesis, and enzymatic assays. From the point of view of structural biology (conformational characteristics of IDO1), it was found that helices constituting the allosteric site did not undergo significant modifications; the same happened for the JK loop (located at the entrance of the catalytic site). In order to investigate, if the presence of the identified allosteric binders could weaken the binding of orthosteric binders (O_2_, tryptophan), as well as weaken the binding of the heme cofactor, further simulations, involving multiscale quantum mechanics/molecular mechanics (QM/MM) are being performed in the laboratory.

## Figures and Tables

**Table 1:** Structure-based (second) virtual screening.

Compound	Docking (kcal/mol)	Compound	Docking (kcal/mol)
chamuvaritin	−7.9	Kushenol C	−6.5
**chamaejasmin B**	**−7.9**	butin	−6.5
**dichamanetin**	**−7.8**	estrobopinin	−6.4
chamaejasmin	−7.7	rhamnocitrin	−6.4
neochamaejasmin A	−7.6	7-benziloxicumarin	−6.3
obovatine	−7.5	naringenin	−6.3
**isochamanetin**	**−7.5**	uvaretin	−6.2
β-naftoflavone	−6.9	pinocembrin	−6.1
pinobanksine	−6.9	genkwanin	−6.1
tectocrisina	−6.8	glabranin	−6.0
soforaflavanone B	−6.7	7-hidroxiflavanone	−6.0
strobopinin-7-methyl-ether	−6.7	apigenin-4’,7-dimethyl-ether	−6.0
diuvaretin	−6.6	2-hidroxiflavanone	−6.0
izalpinin	−6.6	asebogenine	−5.9
**Kushenol E**	**−6.6**	steppogenin	−5.8

**Table 2:** The interacting amino acid residues of IDO1 with chamaejasmin B, dichamanetin, isochamanetin, Kushenol E, and steppogenin.

Compound	Molecular Structure	H Bond	van der Waals	Pi-Alkyl	Pi-Sigma	Pi-Pi	Alkyl	Unfavorable
chamaejasmin B	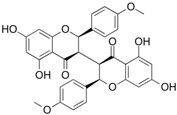	Phe185, Asn313	Tyr15, Ile181, Gln189, Met188, Phe306, Ser309, Leu310	Pro182, Pro314	-	Phe185	Lys186, Pro182	-
dichamanetin	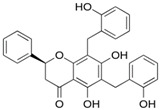	Ser12, Gly11	Ala13, Tyr15, Lys179, Ile181, Lys186, Gln189, Phe306, Ser309, Leu310, Asn313, Pro314	Pro182	Pro182	Phe185	-	-
isochamanetin	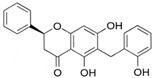	Ser12,	Gly11, Ala13, Lys179, Lys186, Gln189, Asn313, Pro314	Pro182	Pro182	Phe185	-	-
Kushenol E	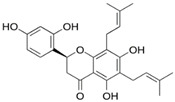	Ser12	Gly11, Lys179, Phe185, Lys186, Gln189, Asn313, Pro314	Pro182	Pro182	-	Ile181, Pro182, Phe306, Leu310	-
steppogenin	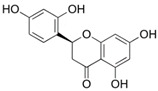	Asn313	Ser12, Tyr15, Phe185, Lys186, Phe306, Ser309, Leu310, Pro314	Pro182	-	-	-	Gln189

**Table 3:** Free energy of interaction of each flavonoid with IDO1.

Energy Component	Kushenol E ΔG (kcal/mol)	Steppogenin ΔG (kcal/mol)	Dichamanetin ΔG (kcal/mol)	Isochamanetin ΔG (kcal/mol)
van der Waals	−33.4464 +/− 2.7222	−12.6589 +/− 5.4591	−38.7571 +/− 2.8011	−33.1692 +/− 2.6773
Electrostatic	−0.4995 +/− 2.3494	−9.5176 +/− 5.6553	−8.1238 +/− 3.5253	−2.7772 +/− 2.1816
Polar Solvation	16.2786 +/− 3.5898	18.1821 +/− 8.5497	25.4326 +/− 4.2624	17.7325 +/− 2.3312
SASA	−3.9866 +/− 0.2390	−1.7428 +/− 0.7148	−4.4861 +/− 0.2483	−3.8790 +/− 0.3585
Free energy of interaction	−21.6563 +/− 3.1142	−5.7373 +/− 5.2005	−25.9345 +/− 3.1214	−22.0960 +/− 3.1859

**Table 4:** Distributed binding energies per amino acid for each modeled IDO1-flavonoid complex.

Alloesteric Site	Kushenol E ΔG (kcal/mol)	Steppogenin ΔG (kcal/mol)	Dichamanetin ΔG (kcal/mol)	Isochamanetin ΔG (kcal/mol)
Lys179	−5.6572 +/− 0.009	0.0522 +/− 0.0146	−10.2480 +/− 0.0138	−0.1242 +/− 0.0023
Ile181	−5.5043 +/− 0.003	−0.0478 +/− 0.0006	−7.1328 +/− 0.0045	−5.9646 +/− 0.0042
Pro182	−2.7174 +/− 0.009	−0.6856 +/− 0.0075	−2.9304 +/− 0.0094	−0.9383 +/− 0.0058
Phe185	−9.7036 +/− 0.0064	−0.1037 +/− 0.0017	−10.9729 +/− 0.0063	−2.6943 +/− 0.0152
Lys186	−0.0069 +/− 0.0271	−0.6023 +/− 0.0201	−12.8116 +/− 0.0194	−3.0506 +/− 0.0093
Met188	−0.1075 +/− 0.0007	−0.0451 +/− 0.0006	−0.1199 +/− 0.0006	−6.0922 +/− 0.0049
Gln189	−4.2292 +/− 0.0045	−0.0770 +/− 0.0020	−4.7260 +/− 0.0061	−7.7712 +/− 0.0108
Phe306	−13.3145 +/− 0.0084	−0.0055 +/− 0.0003	−14.2844 +/− 0.0076	−7.4103 +/− 0.0070
Ser309	0.0639 +/− 0.0090	0.0004 +/− 0.0012	0.1190 +/− 0.0077	2.6300 +/− 0.0092
Leu310	−6.0265 +/− 0.0047	−0.0027 +/− 0.0004	−6.1063 +/− 0.0043	−6.0442 +/− 0.0048
Asn313	3.2576 +/− 0.0086	−0.0099 +/− 0.0015	−0.0466 +/− 0.0065	−0.0041 +/− 0.0150
Pro314	−0.0440 +/− 0.0013	−0.0007 +/− 0.0007	−0.0231 +/− 0.0009	−5.5250 +/− 0.0143

## Data Availability

Data is contained within the article and supplementary material.

## Supplementary Materials

The following supporting information can be downloaded at: https://www.mdpi.com/article/10.3390/ph15091099/s1, Figure S1: Radius of gyration plots of apo IDO1 (black), IDO1-Kushenol E (red), IDO1-steppogenin (magenta), IDO1-dichamanetin (blue), and IDO1-isochamanetin (yellow) systems.; Figure S2: RMSD plots. Apo IDO1 (black), IDO1- Kushenol E (red), IDO1-steppogenin (magenta), IDO1-dichamanetin (blue), and IDO1-isochamanetin (yellow); Figure S3: RMSF plots. Apo IDO1 (black), IDO1- Kushenol E (red), IDO1-steppogenin (magenta), IDO1-dichamanetin (blue), and IDO1-isochamanetin (yellow); Figure S4: RMSF plots for the flavonoids: Kushenol E (red), steppogenin (magenta), dichamanetin (blue), and isochamanetin (yellow); Figure S5: Hydrogen bond frequency. IDO1 Kushenol E (red), IDO1-steppogenin (magenta), IDO1-dichamanetin (blue) and IDO1-isochamanetin (yellow); Table S1: Screened compounds using Mcule and ZINC 15 databases.

## References

[1] Esfahani K., Roudaia L., Buhlaiga N., Del Rincon S.V., Papneja N., Miller W.H. (2020). A review of cancer immunotherapy: From the past, to the present, to the future. Curr. Oncol..

[2] Platten M., Nollen E.A.A., Röhrig U.F., Fallarino F., Opitz C.A. (2019). Tryptophan metabolism as a common therapeutic target in cancer, neurodegeneration and beyond. Nat. Rev. Drug Discov..

[3] Le Floc’h N., Otten W., Merlot E. (2011). Tryptophan metabolism, from nutrition to potential therapeutic applications. Amino Acids.

[4] Lanser L., Kink P., Egger E.M., Willenbacher W., Fuchs D., Weiss G., Kurz K. (2020). Inflammation-Induced Tryptophan Breakdown is Related with Anemia, Fatigue, and Depression in Cancer. Front. Immunol..

[5] Hornyák L., Dobos N., Koncz G., Karányi Z., Páll D., Szabó Z., Halmos G., Székvölgyi L. (2018). The role of indoleamine-2,3-dioxygenase in cancer development, diagnostics, and therapy. Front. Immunol..

[6] Nguyen N.T., Kimura A., Nakahama T., Chinen I., Masuda K., Nohara K., Fujii-Kuriyama Y., Kishimoto T. (2010). Aryl hydrocarbon receptor negatively regulates dendritic cell immunogenicity via a kynurenine-dependent mechanism. Proc. Natl. Acad. Sci. USA.

[7] Pallotta M.T., Fallarino F., Matino D., Macchiarulo A., Orabona C. (2014). AhR-mediated, non-genomic modulation of IDO1 function. Front. Immunol..

[8] Prendergast G.C., Malachowski W.J., Mondal A., Scherle P., Muller A.J. (2018). Indoleamine 2,3-Dioxygenase and Its Therapeutic Inhibition in Cancer. Int. Rev. Cell Mol. Biol..

[9] Vogel C.F.A., Goth S.R., Dong B., Pessah I.N., Matsumura F. (2008). Aryl hydrocarbon receptor signaling mediates expression of indoleamine 2,3-dioxygenase. Biochem. Biophys. Res. Commun..

[10] Hascitha J., Priya R., Jayavelu S., Dhandapani H., Selvaluxmy G., Sunder Singh S., Rajkumar T. (2016). Analysis of Kynurenine/Tryptophan ratio and expression of IDO1 and 2 mRNA in tumour tissue of cervical cancer patients. Clin. Biochem..

[11] Löb S., Königsrainer A., Zieker D., Brücher B.L.D.M., Rammensee H.G., Opelz G., Terness P. (2009). IDO1 and IDO2 are expressed in human tumors: Levo-but not dextro-_1_-methyl tryptophan inhibits tryptophan catabolism. Cancer Immunol. Immunother..

[12] Theate I., Van Baren N., Pilotte L., Moulin P., Larrieu P., Renauld J.C., Herve C., Gutierrez-Roelens I., Marbaix E., Sempoux C. (2015). Extensive profiling of the expression of the indoleamine 2,3-dioxygenase 1 protein in normal and tumoral human tissues. Cancer Immunol. Res..

[13] Sook Y., Hamdy H. (1995). Involvement of Two Regulatory Elements in Interferon-7-Regulated Expression of Human Indoleamine 2,3-Dioxygenase Gene. J. Interferon Cytokine Res..

[14] Prendergast G.C., Jaffee E.M. (2013). Cancer Immunotherapy: Immune Suppression and Tumor Growth.

[15] Mirgaux M., Leherte L., Wouters J. (2020). Influence of the presence of the heme cofactor on the JK-loop structure in indoleamine 2,3-dioxygenase 1. Acta Crystallogr. Sect. D: Struct. Biol..

[16] Liu X., Zhang Y., Duan H., Luo Q., Liu W., Liang L., Wan H., Chang S., Hu J., Shi H. (2020). Inhibition Mechanism of Indoleamine 2, 3-Dioxygenase 1 (IDO1) by Amidoxime Derivatives and Its Revelation in Drug Design: Comparative Molecular Dynamics Simulations. Front. Mol. Biosci..

[17] Lewis-Ballester A., Pham K.N., Batabyal D., Karkashon S., Bonanno J.B., Poulos T.L., Yeh S.R. (2017). Structural insights into substrate and inhibitor binding sites in human indoleamine 2,3-dioxygenase 1. Nat. Commun..

[18] Nelp M.T., Kates P.A., Hunt J.T., Newitt J.A., Balog A., Maley D., Zhu X., Abell L., Allentoff A., Borzilleri R. (2018). Immune-modulating enzyme indoleamine 2,3-dioxygenase is effectively inhibited by targeting its apo-form. Proc. Natl. Acad. Sci. USA.

[19] Röhrig U.F., Reynaud A., Majjigapu S.R., Vogel P., Pojer F., Zoete V. (2019). Inhibition Mechanisms of Indoleamine 2,3-Dioxygenase 1 (IDO1). J. Med. Chem..

[20] Opitz C.A., Litzenburger U.M., Opitz U., Sahm F., Ochs K., Lutz C., Wick W., Platten M. (2011). The indoleamine-2,3-dioxygenase (IDO) inhibitor 1-methyl-d-tryptophan upregulates IDO1 in human cancer cells. PLoS ONE.

[21] Wang N., Zhang J., Li Q., Xu H., Chen G., Li Z., Liu D., Yang X. (2019). Discovery of potent indoleamine 2,3-dioxygenase (IDO) inhibitor from alkaloids in *Picrasma quassioides* by virtual screening and in vitro evaluation. Fitoterapia.

[22] Yue E.W., Sparks R., Polam P., Modi D., Douty B., Wayland B., Glass B., Takvorian A., Glenn J., Zhu W. (2017). INCB24360 (Epacadostat), a Highly Potent and Selective Indoleamine-2,3-dioxygenase 1 (IDO1) Inhibitor for Immuno-oncology. ACS Med. Chem. Lett..

[23] Ortiz-Meoz R.F., Wang L., Matico R., Rutkowska-Klute A., De la Rosa M., Bedard S., Midgett R., Strohmer K., Thomson D., Zhang C. (2021). Characterization of Apo-Form Selective Inhibition of Indoleamine 2,3-Dioxygenase. ChemBioChem.

[24] Kwon M., Ko S.K., Jang M., Kim G.H., Ryoo I.J., Son S., Ryu H.W., Oh S.R., Lee W.K., Kim B.Y. (2019). Inhibitory effects of flavonoids isolated from Sophora flavescens on indoleamine 2,3-dioxygenase 1 activity. J. Enzym. Inhib. Med. Chem..

[25] Kiss R., Sandor M., Szalai F.A. (2012). http://Mcule.com: A public web service for drug discovery. J. Cheminformatics.

[26] Sterling T., Irwin J.J. (2015). ZINC 15-Ligand Discovery for Everyone. J. Chem. Inf. Modeling.

[27] Tanimoto T.T. (1958). Elementary Mathematical Theory of Classification and Prediction.

[28] Chokchaisiri R., Kunkaewom S., Chokchaisiri S., Ganranoo L., Chalermglin R., Suksamrarn A. (2017). Potent cytotoxicity against human small cell lung cancer cells of the heptenes from the stem bark of Xylopia pierrei Hance. Med. Chem. Res..

[29] Quimque M.T., Notarte K.I., Letada A., Fernandez R.A., Pilapil D.Y., Pueblos K.R., Agbay J.C., Dahse H.M., Wenzel-Storjohann A., Tasdemir D. (2021). Potential Cancer- And Alzheimer’s Disease-Targeting Phosphodiesterase Inhibitors from Uvaria alba: Insights from in Vitro and Consensus Virtual Screening. ACS Omega.

[30] Yong Y., Matthew S., Wittwer J., Pan L., Shen Q., Kinghorn A.D., Swanson S.M., Blanco E.J.C.D. (2013). Dichamanetin Inhibits Cancer Cell Growth by Affecting ROS-related Signaling Components through Mitochondrial-mediated Apoptosis. Anticancer. Res..

[31] Swarnalatha Y., Vidhya V.G., Murugan A. (2019). Isochamanetin is a Selective Inhibitor for CyclinD1 in SKOV3 Cell Lines. Nutr. Cancer.

[32] Lasswell W.L., Hufford C.D. (1976). Cytotoxic C-Benzylated Flavonoids from Uvaria chamae. J. Org. Chem..

[33] Zhang C., Zhou S.S., Feng L.Y., Zhang D.Y., Lin N.M., Zhang L.H., Pan J.P., Wang J.B., Li J. (2013). In vitro anti-cancer activity of chamaejasmenin B and neochamaejasmin C isolated from the root of *Stellera chamaejasme* L.. Acta Pharmacol. Sin..

[34] Zondlo N.J. (2012). Aromatic-Proline Interactions: Electronically Tunable CH/π Interactions. Acc. Chem. Res..

[35] Martinez C.R., Iverson B.L. (2012). Rethinking the term “pi-stacking”. Chem. Sci..

[36] Berman H.M., Westbrook J., Feng Z., Gilliland G., Bhat T.N., Weissig H., Shindyalov I.N., Bourne P.E. (2000). The Protein Data Bank. Nucleic Acids Res..

[37] Bayly C.I., Cieplak P., Cornell W.D., Kollman P.A. (1993). A well-behaved electrostatic potential based method using charge restraints for deriving atomic charges: The RESP model. J. Phys. Chem..

[38] Gasteiger J., Marsili M. (1980). Iterative partial equalization of orbital electronegativity-a rapid access to atomic charges. Tetrahedron.

[39] Trott O., Olson A.J. (2009). AutoDock Vina: Improving the speed and accuracy of docking with a new scoring function, efficient optimization, and multithreading. J. Comput. Chem..

[40] Huang J., Rauscher S., Nawrocki G., Ran T., Feig M., De Groot B.L., Grubmüller H., MacKerell A.D. (2016). CHARMM36m: An improved force field for folded and intrinsically disordered proteins. Nat. Methods.

[41] Abraham M.J., Murtola T., Schulz R., Páll S., Smith J.C., Hess B., Lindah E. (2015). Gromacs: High performance molecular simulations through multi-level parallelism from laptops to supercomputers. SoftwareX.

[42] Vanommeslaeghe K., Hatcher E., Acharya C., Kundu S., Zhong S., Shim J., Darian E., Guvench O., Lopes P., Vorobyov I. (2010). CHARMM General Force Field (CGenFF): A force field for drug-like molecules compatible with the CHARMM all-atom additive biological force fields. J. Comput. Chem..

[43] Berendsen H.J.C., Postma J.P.M., Van Gunsteren W.F., Dinola A., Haak J.R. (1984). Molecular dynamics with coupling to an external bath. J. Chem. Phys..

[44] Parrinello M., Rahman A. (1981). Polymorphic transitions in single crystals: A new molecular dynamics method. J. Appl. Phys..

[45] Essmann U., Perera L., Berkowitz M.L., Darden T., Lee H., Pedersen L.G. (1995). A smooth particle mesh Ewald method. J. Chem. Phys..

[46] Kumari R., Kumar R., Lynn A. (2014). G-mmpbsa-A GROMACS tool for high-throughput MM-PBSA calculations. J. Chem. Inf. Modeling.

[47] (2021). BIOVIA, Dassault Systèmes.

[48] Pettersen E.F., Goddard T.D., Huang C.C., Couch G.S., Greenblatt D.M., Meng E.C., Ferrin T.E. (2004). UCSF Chimera—a visualization system for exploratory research and analysis. J. Comput. Chem..

[49] Morris G.M., Ruth H., Lindstrom W., Sanner M.F., Belew R.K., Goodsell D.S., Olson A.J. (2009). Software news and updates AutoDock4 and AutoDockTools4: Automated docking with selective receptor flexibility. J. Comput. Chem..

[50] Grace. https://plasma-gate.weizmann.ac.il/Grace/.

