# Supplementary material for: Identification of Potential Allosteric Site Binders of Indoleamine 2,3-Dioxygenase 1 from Plants: A Virtual and Molecular Dynamics Investigation
Source: Pharmaceuticals (Basel). 2022 Sep 2;15(9):1099. doi: 10.3390/ph15091099 (PMC9502501; doi:10.3390/ph15091099)
Supplement: Supplementary file 1 [file pharmaceuticals-15-01099-s001.zip › pharmaceuticals-1839372-supplementary.pdf]

*pharmaceuticals*

*Article*

# Identification of Potential Allosteric Site Binders of Indoleamine 2,3-Dioxygenase 1 from Plants: A Virtual and Molecular Dynamics Investigation

**Vitor Martins de Almeida [1], Osvaldo Andrade Santos-Filho [1,*]**

[1] Laboratório de Modelagem Molecular e Biologia Estrutural Computacional, Instituto de Pesquisas de Produtos Naturais Walter Mors, Centro de Ciências da Saúde, Universidade Federal do Rio de Janeiro, Av. Carlos Chagas Filho, 373, Bloco H, 21941-599, Cidade Universitária, Rio de Janeiro, RJ, Brazil

* Correspondence: osvaldo@ippn.ufrj.br

Received: date; Accepted: date; Published: date

## Supplementary Material

**Table S1.** Screened compounds using Mcule and ZINC 15 databases. The reference compound (Kushenol E) is marked in bold black. The 19 screened compounds used in the second-round docking simulations are shown in red.

| Compound (ID code) | Docking (kcal/mol) |
|---|---|
| MCULE-5576692992-0 | -8,1 |
| MCULE-9555611424-0 | -8 |
| MCULE-2245505834-0 | -7,8 |
| MCULE-1714548327-0 | -7,7 |
| MCULE-8275106243-0 | -7,6 |
| MCULE-6303182626-0 | -7,6 |
| MCULE-8135607815-0 | -7,6 |
| MCULE-9361233668-0 | -7,6 |
| MCULE-2627819626-0 | -7,5 |
| MCULE-5577419993-0 | -7,5 |
| MCULE-3109336878-0 | -7,4 |
| MCULE-4057380396-0 | -7,4 |
| MCULE-1477744891-0 | -7,4 |
| MCULE-7842380240-0 | -7,3 |
| MCULE-3613127722-0 | -7,2 |
| MCULE-2530592174-0 | -7,2 |
| MCULE-6290700428-0 | -7,1 |

| Compound (ID code) | Docking (kcal/mol) |
|---|---|
| ZINC000014727588 | -6,7 |
| **Kushenol E** | **-6,6** |
| MCULE-7258052902-0 | -6,6 |
| MCULE-6668187578-0 | -6,6 |
| MCULE-5457759324-0 | -6,6 |
| MCULE-2108662486-0 | -6,6 |
| ZINC000059587549 | -6,6 |
| ZINC000013485407 | -6,6 |
| MCULE-6745304759-0 | -6,5 |
| MCULE-8662675920-0 | -6,5 |
| MCULE-7337415562-0 | -6,5 |
| MCULE-8223070696-0 | -6,5 |
| ZINC000003873159 | -6,5 |
| ZINC000014780759 | -6,5 |
| ZINC000014727587 | -6,5 |
| ZINC000003871182 | -6,5 |
| MCULE-7064559962-0 | -6,4 |

| | |
|---|---|
| MCULE-9612591345-0 | -7,1 |
| MCULE-3904196669-0 | -7,1 |
| MCULE-6814312961-0 | -7 |
| ZINC000013339838 | -7 |
| ZINC000034165799 | -7 |
| MCULE-7513860584-0 | -6,9 |
| MCULE-3558918209-0 | -6,9 |
| MCULE-6717818413-0 | -6,9 |
| MCULE-5790553332-0 | -6,9 |
| MCULE-6122035709-0 | -6,9 |
| MCULE-6772374144-0 | -6,9 |
| MCULE-8640003899-0 | -6,9 |
| MCULE-6920051494-0 | -6,9 |
| MCULE-6883102548-0 | -6,9 |
| MCULE-8604836333-0 | -6,8 |
| MCULE-5717525103-0 | -6,8 |
| MCULE-6672284700-0 | -6,8 |
| MCULE-1514405777-0 | -6,8 |
| MCULE-4827438323-0 | -6,7 |
| MCULE-1097395119-0 | -6,7 |
| MCULE-1088177999-0 | -6,7 |
| MCULE-1651216901-0 | -6,7 |
| MCULE-4867351823-0 | -6,7 |
| MCULE-8960596008-0 | -6,7 |
| MCULE-4396859889-0 | -6,7 |
| ZINC000014727515 | -6,7 |
| ZINC000014727518 | -6,7 |
| ZINC000033832203 | -6,7 |
| MCULE-6674323180-0 | -6,2 |
| MCULE-7736830441-0 | -6,2 |
| MCULE-8939875451-0 | -6,2 |
| MCULE-2660644517-0 | -6,2 |
| MCULE-2776613173-0 | -6,2 |
| MCULE-5895803515-0 | -6,2 |
| MCULE-3925941144-0 | -6,2 |
| MCULE-5180733045-0 | -6,2 |
| MCULE-8876698541-0 | -6,2 |
| MCULE-8465457323-0 | -6,2 |
| MCULE-1040954793-0 | -6,2 |
| MCULE-3016034856-0 | -6,2 |

| | |
|---|---|
| MCULE-7607710461-0 | -6,4 |
| MCULE-2373585148-0 | -6,4 |
| MCULE-4528750254-0 | -6,4 |
| MCULE-4861196207-0 | -6,4 |
| MCULE-3082150001-0 | -6,4 |
| MCULE-2990146105-0 | -6,4 |
| MCULE-2154315081-0 | -6,4 |
| ZINC000005158921 | -6,4 |
| ZINC000014641685 | -6,4 |
| MCULE-1335758047-0 | -6,3 |
| MCULE-8645775041-0 | -6,3 |
| MCULE-3559522784-0 | -6,3 |
| MCULE-8531878073-0 | -6,3 |
| MCULE-7707382821-0 | -6,3 |
| MCULE-1736405258-0 | -6,3 |
| ZINC000013485409 | -6,3 |
| ZINC000005854394 | -6,3 |
| MCULE-6580599773-0 | -6,2 |
| MCULE-4547911471-0 | -6,2 |
| MCULE-4427780251-0 | -6,2 |
| MCULE-1272280425-0 | -6,2 |
| MCULE-2862082028-0 | -6,2 |
| MCULE-7196504979-0 | -6,2 |
| MCULE-9501093013-0 | -6,2 |
| MCULE-5533647840-0 | -6,2 |
| MCULE-2337043419-0 | -6,2 |
| MCULE-7830277688-0 | -6,2 |
| MCULE-1466545273-0 | -6,2 |
| MCULE-7830277688-0 | -6,2 |
| MCULE-7439794905-0 | -6,1 |
| MCULE-8195585327-0 | -6 |
| MCULE-1893775486-0 | -6 |
| MCULE-6964743359-0 | -6 |
| MCULE-9439004515-0 | -6 |
| MCULE-7578970975-0 | -6 |
| MCULE-8498690406-0 | -6 |
| MCULE-5306878717-0 | -6 |
| MCULE-2064774552-0 | -6 |
| MCULE-5637234780-0 | -6 |
| MCULE-7275500281-0 | -6 |

| | |
|---|---|
| MCULE-6590579030-0 | -6,2 |
| MCULE-1122459209-0 | -6,2 |
| MCULE-8240919679-0 | -6,2 |
| MCULE-2424476488-0 | -6,2 |
| MCULE-6451111457-0 | -6,2 |
| MCULE-5001672302-0 | -6,1 |
| MCULE-9917900371-0 | -6,1 |
| MCULE-9197350217-0 | -6,1 |
| MCULE-7104867839-0 | -6,1 |
| MCULE-5704405221-0 | -6,1 |
| MCULE-7416386512-0 | -6,1 |
| MCULE-2598470840-0 | -6,1 |
| MCULE-6753110271-0 | -6,1 |
| MCULE-3240859900-0 | -6,1 |
| MCULE-1297538743-0 | -6,1 |
| MCULE-5852778653-0 | -6,1 |
| MCULE-3254872691-0 | -6,1 |
| MCULE-3976263760-0 | -6,1 |
| MCULE-5052743999-0 | -6,1 |
| MCULE-9302131889-0 | -6,1 |
| MCULE-9370973230-0 | -6,1 |
| MCULE-6631561492-0 | -6,1 |
| MCULE-8726416131-0 | -6,1 |
| MCULE-8084114235-0 | -6,1 |
| MCULE-3924606015-0 | -6,1 |
| MCULE-4114823523-0 | -6,1 |
| MCULE-6331583005-0 | -6,1 |
| MCULE-1517499241-0 | -6,1 |
| MCULE-4015613387-0 | -6,1 |
| MCULE-3938369351-0 | -6,1 |
| MCULE-3625538935-0 | -6,1 |

| | |
|---|---|
| MCULE-9332914030-0 | -6 |
| MCULE-7826903505-0 | -6 |
| MCULE-6305563775-0 | -6 |
| MCULE-8743190189-0 | -6 |
| MCULE-1243164622-0 | -6 |
| MCULE-4107309014-0 | -5,9 |
| MCULE-5489217450-0 | -5,9 |
| MCULE-2527326110-0 | -5,9 |
| MCULE-4005429744-0 | -5,9 |
| MCULE-8068976578-0 | -5,9 |
| MCULE-1231893585-0 | -5,9 |
| MCULE-2855478624-0 | -5,9 |
| MCULE-5357027007-0 | -5,9 |
| MCULE-5732404629-0 | -5,9 |
| MCULE-6016729456-0 | -5,9 |
| MCULE-7442958115-0 | -5,8 |
| MCULE-7259979801-0 | -5,8 |
| MCULE-9865914914-0 | -5,8 |
| MCULE-6698275589-0 | -5,8 |
| MCULE-2067187231-0 | -5,8 |
| MCULE-2864630695-0 | -5,7 |
| MCULE-1075981976-0 | -5,7 |
| MCULE-9497505068-0 | -5,7 |
| MCULE-8143016945-0 | -5,7 |
| MCULE-2924473141-0 | -5,6 |
| MCULE-9138980318-0 | -5,6 |
| MCULE-6894860287-0 | -5,5 |
| MCULE-1219453843-0 | -5,5 |
| MCULE-4510679729-0 | -5,4 |
| MCULE-1746821853-0 | -5,3 |

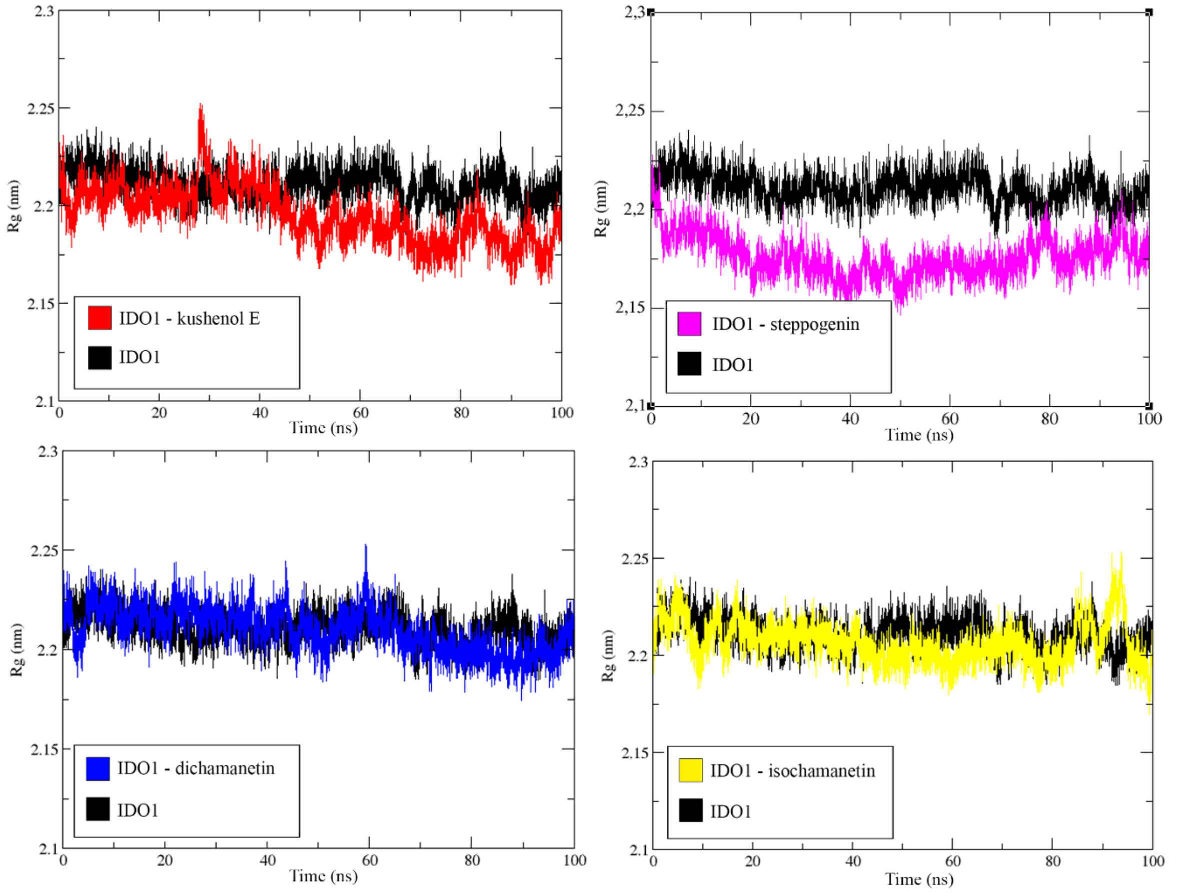


**Figure S1.** Radius of gyration plots of apo IDO1 (black), IDO1-Kushenol E (red), IDO1-steppogenin (magenta), IDO1-dichamanetin (blue), and IDO1-isochamanetin (yellow) systems.

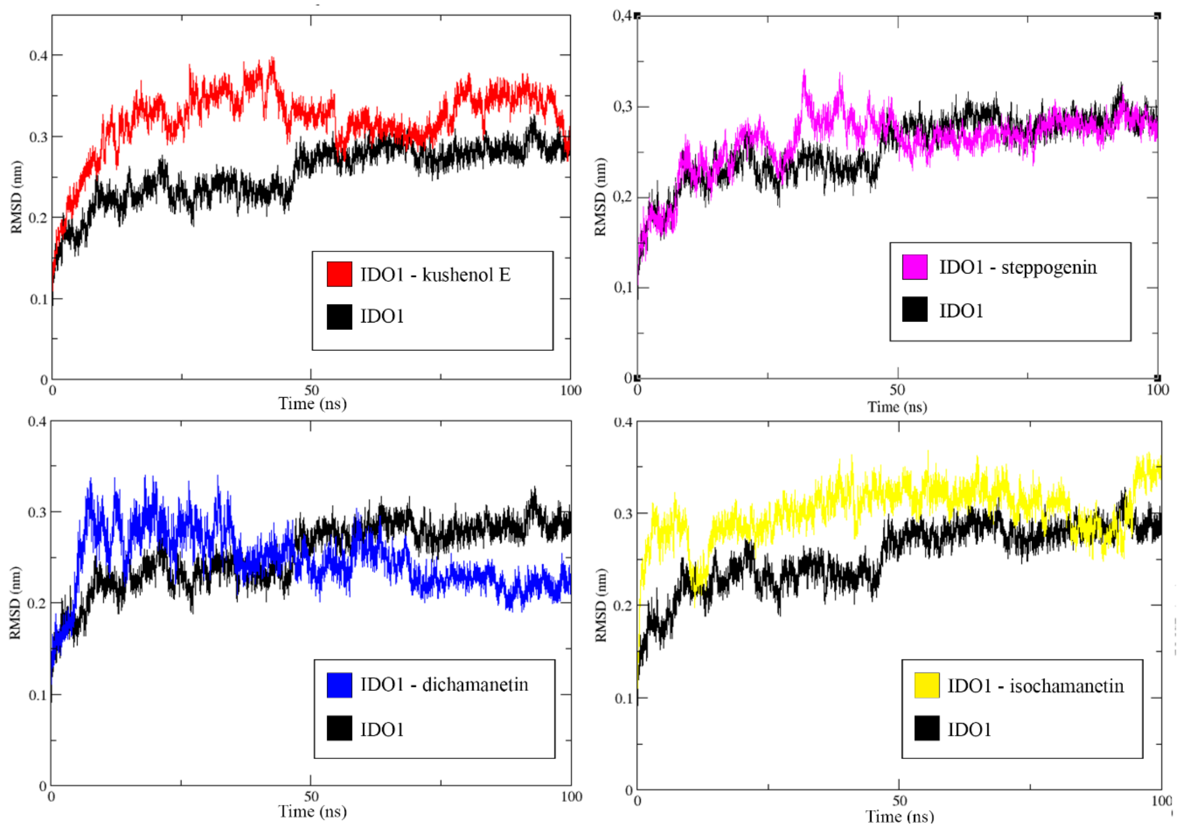


**Figure S2.** RMSD plots. Apo IDO1 (black), IDO1- Kushenol E (red), IDO1-steppogenin (magenta), IDO1-dichamanetin (blue), and IDO1-isochamanetin (yellow).

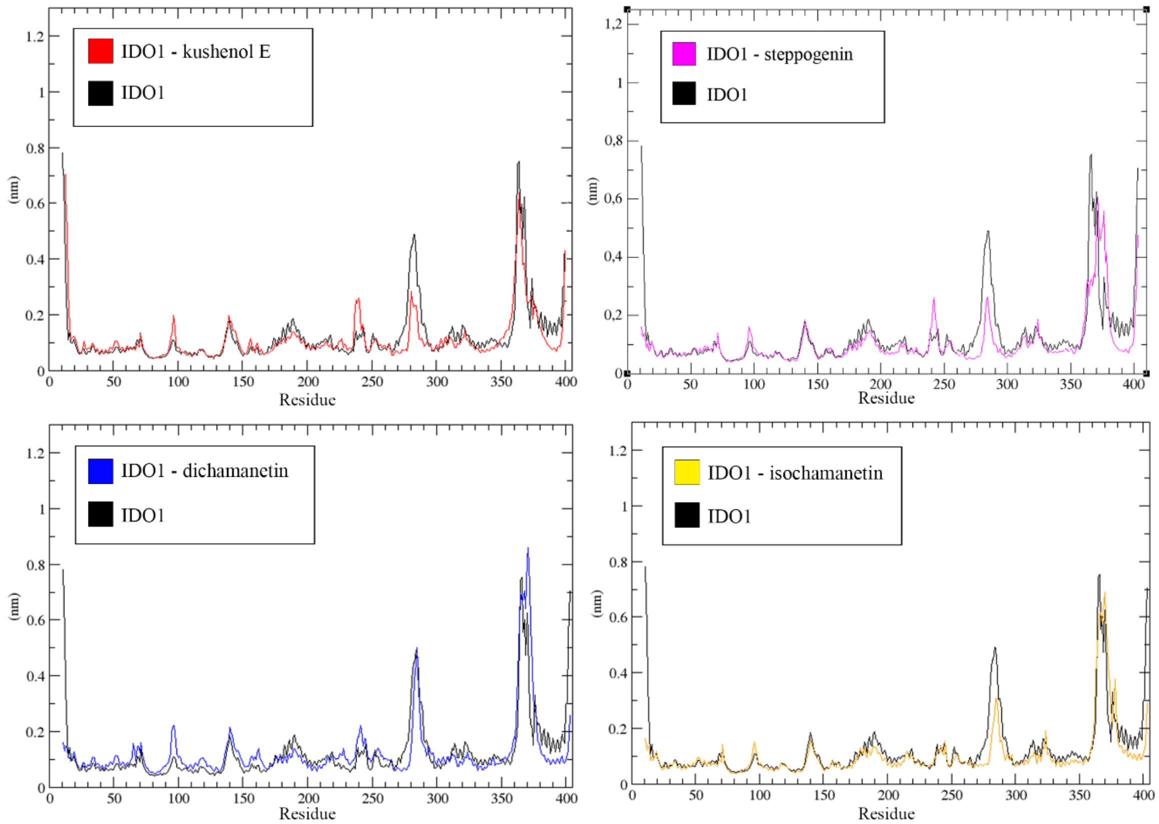


**Figure S3.** RMSF plots. Apo IDO1 (black), IDO1- Kushenol E (red), IDO1-steppogenin (magenta), IDO1-dichamanetin (blue), and IDO1-isochamanetin (yellow).

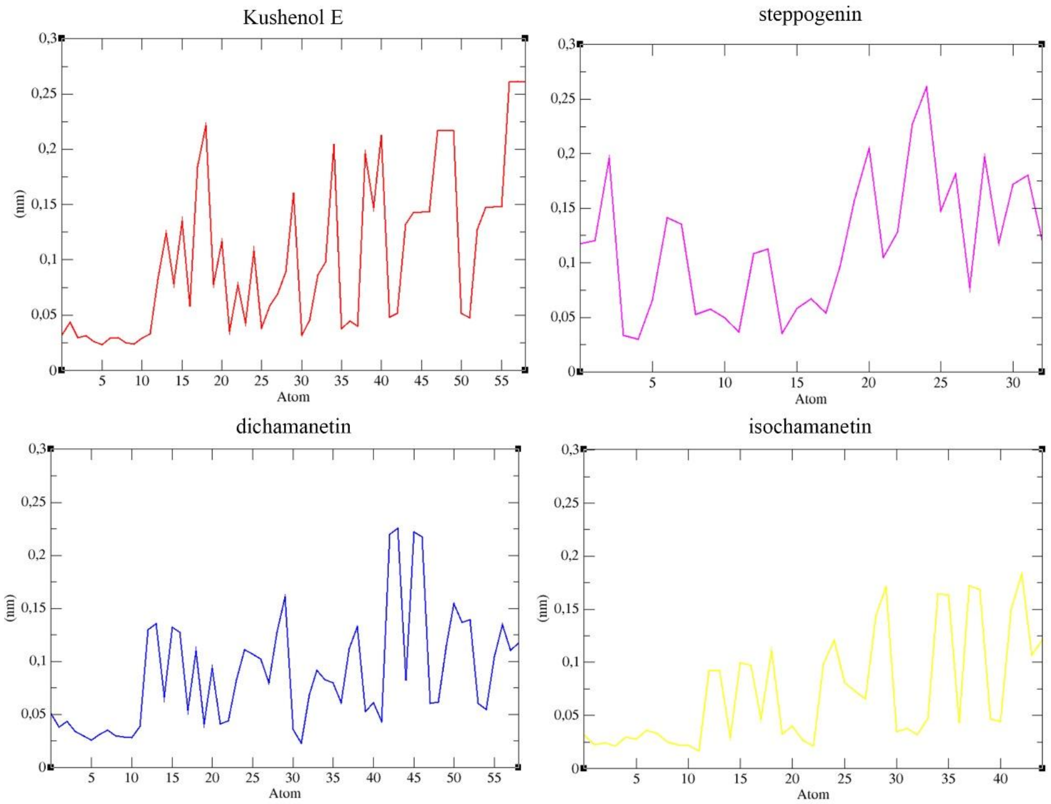


**Figure S4.** RMSF plots for the flavonoids: Kushenol E (red), steppogenin (magenta), dichamanetin (blue), and isochamanetin (yellow).

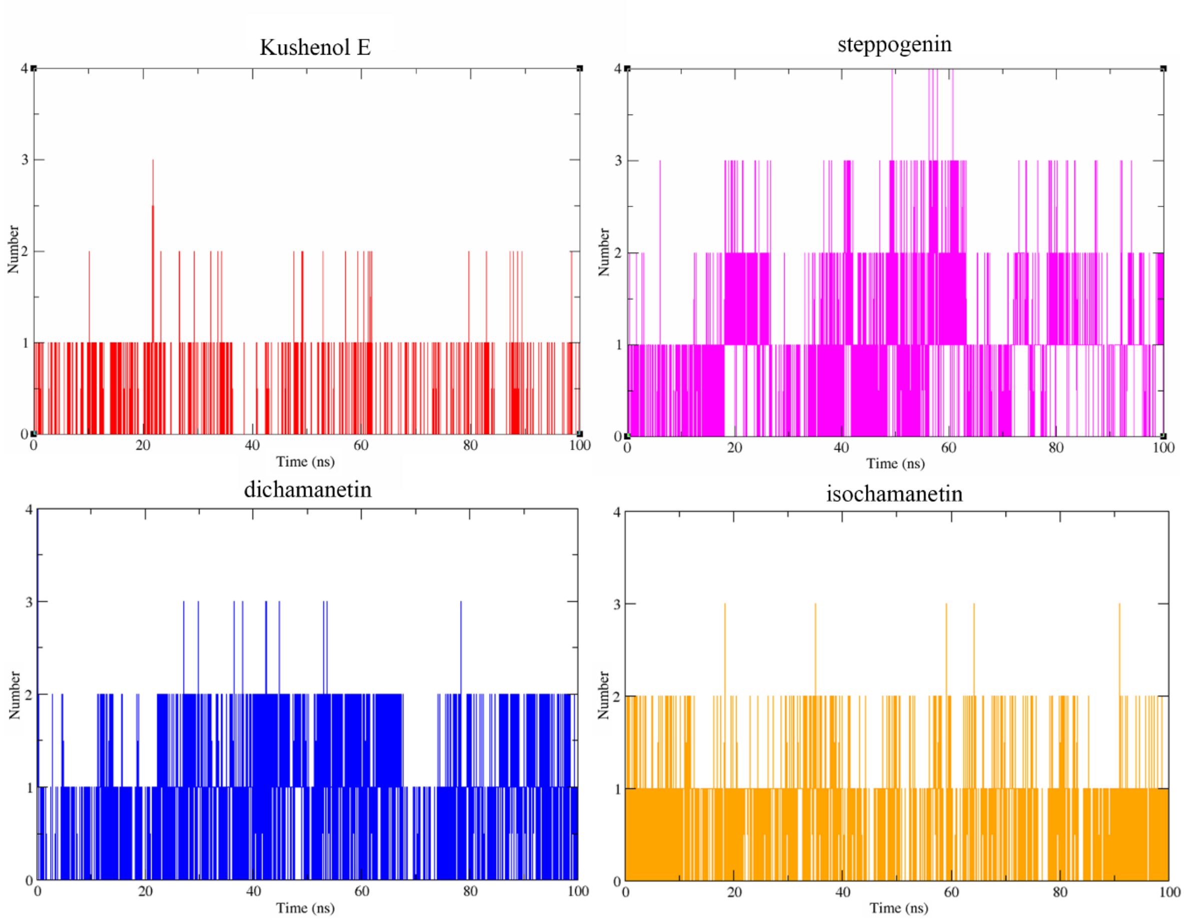


**Figure S5.** Hydrogen bond frequency. IDO1 Kushenol E (red), IDO1-steppogenin (magenta), IDO1-dichamanetin (blue) and IDO1-isochamanetin (yellow).

**Publisher's Note:** MDPI stays neutral with regard to jurisdictional claims in published maps and institutional affiliations.

© 2020 by the authors. Submitted for possible open access publication under the terms and conditions of the Creative Commons Attribution (CC BY) license (http://creativecommons.org/licenses/by/4.0/).
